# Genetic Architecture of Flooding Tolerance in the Dry Bean Middle-American Diversity Panel

**DOI:** 10.3389/fpls.2017.01183

**Published:** 2017-07-06

**Authors:** Ali Soltani, Samira MafiMoghaddam, Katelynn Walter, Daniel Restrepo-Montoya, Sujan Mamidi, Stephan Schroder, Rian Lee, Phillip E. McClean, Juan M. Osorno

**Affiliations:** ^1^Department of Plant Sciences, North Dakota State UniversityFargo, ND, United States; ^2^Genome Sequencing Center, HudsonAlpha Institute for BiotechnologyHuntsville, AL, United States

**Keywords:** common bean, flooding, abiotic stress, anoxia, waterlogging, GWAS

## Abstract

Flooding is a devastating abiotic stress that endangers crop production in the twenty-first century. Because of the severe susceptibility of common bean (*Phaseolus vulgaris* L.) to flooding, an understanding of the genetic architecture and physiological responses of this crop will set the stage for further improvement. However, challenging phenotyping methods hinder a large-scale genetic study of flooding tolerance in common bean and other economically important crops. A greenhouse phenotyping protocol was developed to evaluate the flooding conditions at early stages. The Middle-American diversity panel (*n* = 272) of common bean was developed to capture most of the diversity exits in North American germplasm. This panel was evaluated for seven traits under both flooded and non-flooded conditions at two early developmental stages. A subset of contrasting genotypes was further evaluated in the field to assess the relationship between greenhouse and field data under flooding condition. A genome-wide association study using ~150 K SNPs was performed to discover genomic regions associated with multiple physiological responses. The results indicate a significant strong correlation (*r* > 0.77) between greenhouse and field data, highlighting the reliability of greenhouse phenotyping method. Black and small red beans were the least affected by excess water at germination stage. At the seedling stage, pinto and great northern genotypes were the most tolerant. Root weight reduction due to flooding was greatest in pink and small red cultivars. Flooding reduced the chlorophyll content to the greatest extent in the navy bean cultivars compared with other market classes. Races of Durango/Jalisco and Mesoamerica were separated by both genotypic and phenotypic data indicating the potential effect of eco-geographical variations. Furthermore, several loci were identified that potentially represent the antagonistic pleiotropy. The GWAS analysis revealed peaks at Pv08/1.6 Mb and Pv02/41 Mb that are associated with root weight and germination rate, respectively. These regions are syntenic with two QTL reported in soybean (*Glycine max* L.) that contribute to flooding tolerance, suggesting a conserved evolutionary pathway involved in flooding tolerance for these related legumes.

## Introduction

Low oxygen diffusion in water, prevents its optimal availability to plant organs in flooded conditions and consequently expose plants to hypoxia (e.g., <20.9% and >0% O_2_ at 20°C) or anoxia (e.g., 0% O_2_ at 20°C). Climate change predictions forecast frequent incidents of flooding that will primarily affect poorly-drained arable farmlands (Bailey-Serres et al., 2012a). This stress will jeopardize food security through yield loss. A study looked at the impact of 78 natural disasters in agriculture across 48 countries and concluded that drought drastically affects livestock, but when it comes to cropland, the effects of storms and flooding are more devastating to the food chain (Food and Agriculture Organization of the United Nations, 2015). In the U.S., excess water resulted in ~ $3.2 billion crop damage in 2011 (Bailey-Serres et al., 2012a). A drastic decline in diffusion of O_2_ and CO_2_ molecules available to the plant from surrounding environments is the most detrimental physiological effect of flooding. This limits ATP synthesis and photosynthesis (Bailey-Serres and Voesenek, 2008). Excess water also causes elevated levels of reactive oxygen species (ROS, Blokhina et al., 2003), cytosol acidification (Fan et al., 1988; Menegus et al., 1991; Felle, 2006), and a decrease in root hydraulic conductivity (Holbrook and Zwieniecki, 2003; Tournaire-Roux et al., 2003).

Oxidative phosphorylation is reduced in excess water conditions, and cells compensate by utilizing ATP and NAD from the glycolysis and fermentation pathways. ATP production through fermentation only produces 2–4 ATP per glucose molecule compared to the 36 ATPs per glucose molecule generated from oxidative phosphorylation (Sauter, 2013). This places stressed plants in an energy crisis situation (Colmer and Voesenek, 2009) where energy allocation is strictly economized to the pathways that allow the plant to survive. Energy allocation under flooding varies among and within species at differing developmental stages (Colmer and Voesenek, 2009).

Flooding can be classified as submergence (submergence of the root and aerial portions) or waterlogging (water standing in the root system). Based on environmental factors, the flooding stress can be transitional to prolonged. According to the type of dominant flooding regime in the environment, different species or even different genotypes of the same species adopt either an avoidance, escape, or quiescence strategy (Colmer and Voesenek, 2009; van Veen et al., 2013). The avoidance strategy allows plants to complete their life cycle between two incidents of flooding. This strategy was observed with red goosefoot (*Chenopodium rubrum* L. *syn. Oxybasis rubra* L. S. Fuentes, Uotila & Borsch), that inhabit former riverbeds (Sman et al., 1992). In the escape strategy, morphological and anatomical changes allow the plant to acquire more oxygen from the surrounding environment and deliver it to internal tissues (Bailey-Serres and Voesenek, 2008; Colmer and Voesenek, 2009). These morphological changes include shoot elongation, and aerenchyma and adventitious root formation. The quiescence strategy suppresses the morphological changes related to the escape strategy and suppresses growth. This strategy depends solely on anaerobic energy production (Bailey-Serres and Voesenek, 2008; Colmer and Voesenek, 2009).

The majority of crops employ either the escape or quiescence strategy to cope with flooding stress. For rice (*Oryza sativa* L.), these strategies are mutually exclusive and cannot be present in the same genotype. From a homeostasis standpoint, plants invest their limited resources in anatomical changes that aerate the internal tissues or elevate anaerobic energy production. During prolonged flooding, the escape strategy is preferred over quiescence, however in transient conditions the reverse is the case (Colmer and Voesenek, 2009).

Tolerance to flooding conditions is the outcome of a complex and multi-dimensional interaction between genes that are belonged to several pathways and gene families (Loreti et al., 2016). One of these includes ethylene responsive element binding factor domain (ERF) transcription factors that are involved in hormonal signal transduction and response to several biotic/abiotic stresses, and metabolism regulation (Nakano et al., 2006). The ERF family is a member of the AP2/ERF superfamily which has a single AP2/ERF domain (Nakano et al., 2006). Groups VII and X ERF family members were particularly highlighted as contributors to flooding tolerance in some cases (Liu et al., 2012; Bailey-Serres et al., 2012a).

Common bean is the most important food legume providing protein, iron, fiber, and nutritional elements for direct human consumption (Messina, 1999). An ancestral bean population split into Middle American and Andean gene pools, and domestication occurred independently in these two gene pools (Mamidi et al., 2014; Schmutz et al., 2014). Each gene pool is separated further into races, based on the eco-geographical differences within each region. The three main races within the Middle American gene pool are Durango, Jalisco, and Mesoamerica. Races Durango and Jalisco have medium size seed (25–40 g 100 seed weight^−1^) and include pinto, great northern, pink, and small red market classes and have recently been considered as a single race (Kwak and Gepts, 2009). Race Mesoamerica consist of small seeded (<25 g 100 seed weight^−1^) black and navy market classes. The Andean gene pool contains large seeds (>40 g 100 seed weight^−1^) and is subdivided into races Nueva Granada, Peru, and Chile. Kidney and cranberry beans are the main two market classes within Nueva Granada race (Singh et al., 2007). Middle American market classes are the most common dry beans that are produced in the northern U.S., particularly in North Dakota, the largest dry bean producer state in the US (Knodel et al., 2016).

Flooding stress is a common constraint in the dry bean industry worldwide, particularly in the northern production regions of the US (Knodel et al., 2016). Therefore, evaluating the genetic architecture of flooding tolerance in dry bean is critical considering the current trends of climate change (Bailey-Serres et al., 2012a,b). Several studies investigated the genetic architecture of flooding tolerance in soybean using a bi-parental QTL analyses to map genetic factors associated with the flooding response. Since common bean and soybean diverged ~19.2 million years ago (Lavin et al., 2005) and share extensive blocks of syntenic regions (McClean et al., 2010), mapping the flooding factors in common bean will enable comparative genomic analyses to determine if the factors controlling the stress are shared within the Phaseoleae lineage.

Only one to five common bean genotypes have been evaluated for flooding tolerance (Nelson et al., 1983; Pociecha, 2013; Rajashekar et al., 2014). Flooding appears to drastically reduce the germination percentage (Rajashekar et al., 2014). Furthermore, significant reductions were reported in leaf area, dry root weight and chlorophyll content in response to flooding (Celik and Turhan, 2013). The tolerance level was detected to vary among genotypes which may highlight the potential variation in physiological responses of common bean germplasm to low oxygen (Celik and Turhan, 2013).

The current study was conducted to (i) evaluate the physiological response of Middle-American genotypes to flooding at early growth stages (ii) assess the reliability of greenhouse phenotyping procedure by correlating the results with the field evaluation and (iii) to describe the genetic architecture underling flooding tolerance. To our knowledge, this is the most comprehensive study addressing flooding tolerance in dry bean, and the results will set the stage for further experiments which ultimately lead to the development of varieties with improved flooding tolerance.

## Materials and methods

### Plant materials

The Middle-American Diversity Panel (MDP; Moghaddam et al., 2016) consists of six market classes including pinto (*n* = 95), great northern (*n* = 35), pink (*n* = 22), small red (*n* = 27), black (*n* = 45), and navy (*n* = 48) beans. This panel captures the diversity of dry bean breeding germplasm in North America since the 1930s and represents nearly all released varieties developed by public sector. Royalty, a purple-podded bean variety developed at University of New Hampshire in 1957 was used as a known tolerant check. This variety was shown to have tolerance to flooding stress based on the previous empirical observations (Duke, 1983; T. Kisha, personal communication).

### Experimental design

Plants were evaluated as a randomized complete block design (RCBD) with a split plot arrangement in which the stress and non-stress conditions were two levels of the main plot, and different genotypes were considered as sub-plots. Four replications and two samples per replication were considered for screening at the seedling stage. Three replications were tested to evaluate germination rate.

### Phenotyping procedure in the greenhouse

A phenotyping protocol was developed to evaluate the panel under flooding experiment under greenhouse conditions. The stress conditions and greenhouse temperature were defined based on seven years' rainfall and temperature records (2008–2014) in dry bean growing season in eastern North Dakota (Fargo). These data indicate that the majority of rainfall coincide with V0 to V2 of dry bean growing stages. Furthermore, the temperature range was between 18 and 20°C at these stages.

#### Germination stage

Ten seeds from each genotype were planted in an autoclaved loamy sand soil (83 sand, 11% silt and 6% clay) obtained from the field. This soil contains 420 lb/A of NO_3_, 21 ppm of phosphorous, and 70 ppm of potassium. To induce a flooding condition, sown seeds were submerged in water for 1 day then the water was drained. The same set of seeds were planted and germinated in a well-drained condition for the non-flooded main plot. After 15 days, the germination rate (GR) was evaluated as the percentage of germinated seeds from 10 sown seeds.

#### Seedling stage

Six seeds from each line were planted in pots filled with the same soil type used for germination study. To prevent the soil from spilling out of the pot, a narrow layer of generic potting soil covered the pot drainage holes. All lines were germinated in well-drained conditions. After germination, two healthy seedlings were retained. At the start of the V2 phenological stage (unfolding the primary leaves), the stress-designated plots were exposed to flooding stress. To mimic the flooding conditions in the stress plots, pots were placed in flat storage containers and filled with water until the water level reached 4–5 cm above the soil level. In this condition, although the root system was completely submerged, the leaves were above water. These pots were kept in flooding conditions for 10 days, while the non-flooding plots were grown under well-drained conditions. Non-flooded plants were regularly watered depending on the greenhouse temperature and soil moisture.

After 10 days, the stress plots were drained. Chlorophyll content and adventitious rate (AR) scores were measured at this date. Chlorophyll content was measured on the primary leaves using a SPAD 502 Chlorophyll Meter. Two measurements were recorded from each plant which resulted in a total of four measurements for each line within each replication. The amount of adventitious roots was visually scored from 0 to 5 for each line. Zero indicates no adventitious root and 5 is the highest adventitious root formation compared with other lines. The whole plant, including the above ground tissues and roots were carefully harvested, washed, stored in paper bags, and dried at 38°C for 2 weeks. After drying, the following traits were measured: total weight (TW, g): dry matter weight of whole plant; shoot weight (SW, g): the above-ground tissues including stem and leaves; root weight (RW, g) and hypocotyl length (HL, cm).

### Phenotyping procedure in the field

To evaluate the response of genotypes to flooding in the field condition, a subset of contrasting genotypes that were previously evaluated at the greenhouse were evaluated in the field in 2016. These genotypes include ten tolerant (Apache, Arapaho, CDC Camino, CDCWM2, Chase, Monterrey, Sawtooth, Sequoia, Sonora, and WinMor) and ten susceptible (Albion, Arthur, Envoy, Huron, HY4181, Mackinac, McHale, Medalist, Nautica, and Seabiskit). Royalty was also included as the tolerant check. The field was located on NDSU campus in Fargo, ND (46° 53′58″ N, 96°48′52.3″ W). The soil in this field contains 2% sand, 45% silt, and 53% clay. The main goal of this step was to assess if the greenhouse results were a reliable reflection of the field condition. Twenty-one genotypes were evaluated in the field using a RCBD with split plot arrangement with three replications. In this design, flooded and non-flooded treatments (main plots) were planted side-by-side and genotypes were considered as sub-plots. Eighty seeds were planted per plot using a four row planter. After plants reached to V2 developmental stage, flooded designated plots were flooded. To induce waterlogging condition in the designated field plot, water was delivered via a sprinkler system on the field until the soil saturation. Meanwhile, non-flooded plots were rainfed. After 10 days of flooding conditions, SPAD indices were measured from three random plants within each plot. survival rate of each plot was visually scored from 0 (all plants dead) to 5 (all plants survived).

### Statistical analysis

Analysis of variance and LS-mean estimation was performed in SAS 9.3 using PROC MIXED. In this model, replications were considered as a random effect, and genotypes and treatments were considered as fixed effects. For each trait, a flooding index was calculated for each genotype by Equation (1).

(1) $$\begin{array}{r} Flooding\;index=\;\frac{Flooding\;value\;-\;non\;flooding\;value}{non\;flooding\;value}\; \end{array}$$

The Pearson phenotypic correlations were calculated and plotted in R (R Development Core Team, 2011) using packages psych and corrplot. Z-scores were calculated to test the significance level of difference between correlation coefficients. Genotypic variance ($\delta_{G}^{2}$) were calculated by the method described by Holland et al. (2003). Principal component analysis (PCA) was estimated in the “stats” package in R, using *prcomp* function. Biplots were constructed using ggbiplot package in R. Factor analysis was performed in SPSS 22.0 (IBM Corp, 2013) using the Varimax method with Kaiser normalization (Kaiser, 1958). Factor loading of 0.34 was defined as the significance cut-off for a sample size of 272 individuals (Hair et al., 1998).

### Genome-wide association study

The GWAS was performed using GAPIT (Lipka et al., 2012). ~150 K SNP markers with MAF ≥ 5% were used in this analysis (Moghaddam et al., 2016). Multiple models were tested for each trait under both flooded and non-flooded condition. These models included: (1) null general linear model, (2) general linear model with fixed effects to control for population structure, (3) univariate unified mixed linear model (Yu et al., 2006) using the “population parameters previously determined” protocol (Zhang et al., 2010) to control for individual relatedness (random effect), and (4) a model controlling for both individual relatedness and population structure. We used the first two principal components to account for population structure (cumulatively controlled ~25% of the variation). The best model was selected based on the lowest mean square deviation (MSD, Mamidi et al., 2011). The final Manhattan plots were created using the *mhtplot*() function from the R package “gap” (Zhao, 2007). The phenotypic variation explained by most significant markers (which was defined as the markers with highest LOD) in the best model was calculated based on the likelihood-ratio-based *R*^2^ ($R_{LR}^{2}$) (Sun et al., 2010) using the genABLE package in R (Aulchenko et al., 2007).

Candidate genes were selected from a ± 200 Kb region centered on the most significant marker. The genes were annotated using the Pfam and PANTHER databases (Thomas et al., 2003; Finn et al., 2015). The promoter region of the candidate genes were analyzed for presence of conserved motif using PlantPAN 2.0 and its default settings (Chow et al., 2016). This analysis was conducted to identify the potential transcription factor recognition sites in the promoter and the potential upstream regulatory mechanism of candidate genes.

### Classifying genotypes into the races

Genotypes were classified into races based on a phylogenetic tree which was constructed using the SNPhylo pipeline (Lee et al., 2014). For this analysis, only markers with minor allele frequency (MAF) of 0.05 or higher were used. Furthermore, to choose the most informative SNPs, only markers with pair-wise linkage disequilibrium (LD) *R*^2^-values of 0.10 or lower with all other markers were considered for tree construction.

### Synteny analysis

To identify macro- and micro-synteny between soybean flooding-related QTL and significant regions in this experiment, the sequences of SNP markers bordering the flooding QTL in soybean (http://soybase.org; Grant et al., 2010) were blasted against the dry bean genome. Significant hits (*E*-values less than 1e-40) were considered as orthologous regions and used in the synteny analysis.

## Results

### Separation of races based on genotypic and phenotypic data

A phylogenetic tree of the MDP was constructed using 6,462 SNPs markers that were in low LD (*R*^2^ ≤ 0.10). Two main clusters were detected (Figure S1). The pinto and great northern genotypes clustered in one clade representing the Durango/Jalisco race complex, while the majority of black and navy beans grouped in the Mesoamerica cluster. The majority of pinks (90%) and small red (70%) genotypes were clustered within the Durango/Jalisco cluster.

The phenotypic responses of seven traits were measured in both non-flooded and flooded conditions (Supplementary Material, Data Sheet 1). The frequency distribution analysis indicated all traits for both races were normally distributed (Figure S2). Significant treatment effects were detected for all traits except hypocotyl length (Table 1). Significant differences were also detected among genotypes across treatments for all the traits. Significant interactions between genotype and treatment were detected for five traits including germination rate, total weight, shoot weight, root weight, and SPAD (Table 1).

**Table 1:** Statistical parameters of seven traits.

**Trait**	**Treatment**							**Genotype**			**Treatment × Genotype**
	***F*-value**	**Mean ± sd**		**Range**		**CV%**		***F*-value**	**Mean ± sd**	**CV%**	***F*-value**
		**Non-flooded**	**Flooded**	**Non-flooded**	**Flooded**	**Non-flooded**	**Flooded**				
Germination rate (%)	151.70^**^	71.87 ± 14.63	14.07 ± 12.95	20.00–100.00	0.00–60.00	20.35	92.07	2.54^***^	43.04 ± 32.21	74.84	1.34^**^
Total weight (g)	151.51^**^	1.19 ± 0.25	0.44 ± 0.11	0.50–1.85	0.21–0.72	21.25	26.51	4.06^***^	0.81 ± 0.42	51.97	1.30^**^
Shoot weight (g)	71.15^**^	0.77 ± 0.16	0.34 ± 0.09	0.33–1.23	0.16–0.53	21.08	25.47	4.08^***^	0.55 ± 0.25	45.64	1.24^**^
Root weight (g)	826.00^***^	0.43 ± 0.11	0.10 ± 0.04	0.18–0.72	0.03–0.26	25.59	38.27	2.83^***^	0.26 ± 0.18	68.25	1.39^***^
Hypocotyl length (cm)	1.97	6.56 ± 1.32	6.91 ± 1.39	3.83–10.2	3.92–11.01	20.14	20.13	9.82^***^	6.73 ± 1.37	20.31	0.59
SPAD index	74.48^**^	46.83 ± 3.55	34.09 ± 9.64	36.64–56.10	5.00–51.47	7.58	28.27	6.54^***^	40.46 ± 9.66	23.88	2.88^***^
Adventitious root^†^	–	–	1.61 ± 0.70	–	0.31–3.75	–	43.78	2.52^***^	–	–	–

** *Significant at 0.01 probability level*.

*** *Significant at 0.001 probability level*.

† *Adventitious root was observed only in flooded condition*.

Mean separation analysis revealed that all seven traits vary between races in both non-flooded and flooded conditions (Table 2). In both conditions, Durango/Jalisco genotypes expressed higher total, shoot, and root weight, longer hypocotyls and adventitious roots, and a higher SPAD index (Table 2). Although the germination rate of race Durango/Jalisco (73.62%) was higher in non-flooded condition, germination rate for race Mesoamerica was higher (17.49%) than Durango/Jalisco under the flooding condition.

**Table 2:** Means of seven traits among races.

	**Non-flooded**		**Flooded**	
	**Durango/Jalisco**	**Mesoamerica**	**Durango/Jalisco**	**Mesoamerica**
Germination rate (%)	73.62a^†^	68.23b	12.70a	17.49b
Total weight (g)	1.29a	1.01b	0.51a	0.33b
Shoot weight (g)	0.82a	0.65b	0.38a	0.25b
Root weight (g)	0.47a	0.35b	0.12a	0.07b
Hypocotyl length (cm)	7.04a	5.78b	7.45a	6.01b
SPAD index	48.21a	44.57b	38.82a	26.11b
Adventitious root^‡^	–	–	1.82a	1.27b

† *Means followed by the same letter in each row are not significantly different at 0.05 probability level*.

‡ *Adventitious root was observed only in flooded condition*.

### Flooding indices

**Figure 1:**
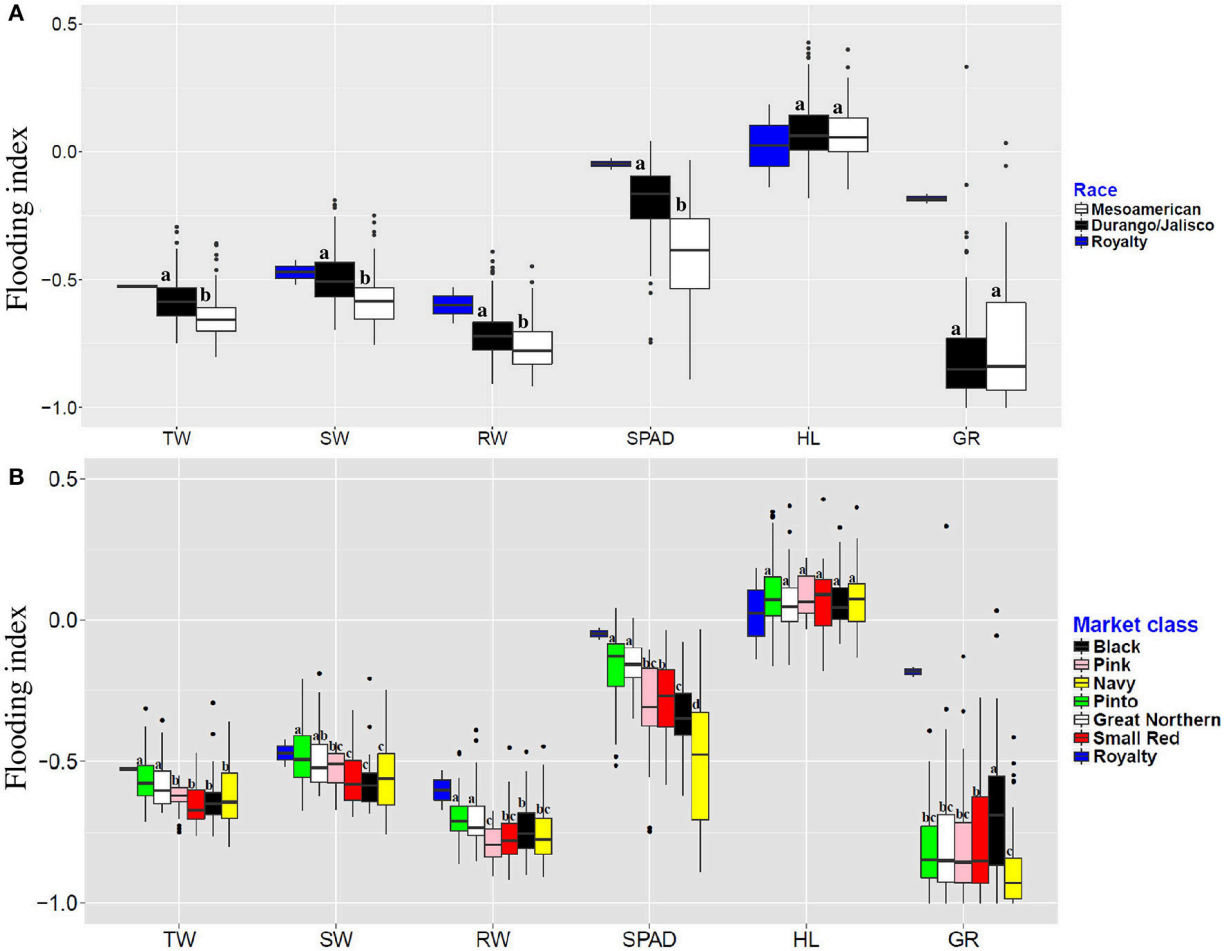
Flooding indices of six traits evaluated in different races **(A)** and market classes **(B)** of dry bean. Means, separated and represented by letters above each boxplot. Groups with the same letter, classified as non-significant difference. TW, Total weight; SW, shoot weight; RW, root weight; HL, hypocotyl length; GR, germination rate.

Flooding indices, which indicates the severity of flooding effect, were calculated for each trait across genotypes. Results showed that the magnitude of flooding indices were the lowest in race Durango/Jalisco at the seedling stage (Figure 1A). Our results also indicate that races and market classes responded differently to the flooding stress (Figure 1B). Royalty, the tolerant check, was the least affected genotype by excess water stress for all traits. The pinto and great northern market classes were least affected by flooding for total weight, shoot weight, root weight, and SPAD index. These two market classes are the most tolerant to flooding stress at the seedling stage. Among the market classes, flooding affected pinks the most for root weight (79% reduction), navy and black beans for chlorophyll content (48 and 34% reduction, respectively), and navy beans for germination rate (84% reduction).

Flooding indices were further used to construct the scatterplots among traits (Figure S3). This analysis revealed outlier genotypes that are potentially using distinct physiological mechanism(s) in response to flooding (Figure S3). For instance, variety Gemini showed highest flooding index (FI = 2.79) for hypocotyl length, although the average FI = 0.09 across other genotypes.

### Principal component and factor analysis

**Figure 2:**
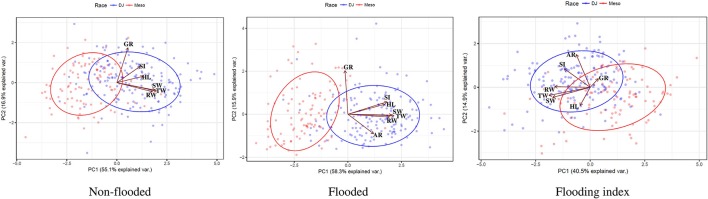
Biplot representation of Principal Component Analysis (PCA), based on phenotypic data in two conditions (non-flooded vs. flooded) and flooding indices. DJ, Durango/Jalisco and Meso; Mesoamerican races highlighted with blue and red, respectively. Vectors indicate the magnitude and direction of loadings.

Principal component analysis was performed separately for non-flooded and flooded conditions as well as flooding indices. For all analyses, races Durango/Jalisco and Mesoamerica clustered at the extreme positions of PC1 (Figure 2). Factor analysis indicated that in both non-flooded and flooded conditions, all traits except germination rate correlated with PC1, and germination rate is the main contributor to the second PC (Figure 2 and Table S1). However, considering the flooding indices, total, shoot and root weight were the main contributors to PC1, and adventitious root and SPAD index were the contributors to PC2 (Figure 2 and Table S1).

### Genotypic variance of traits under both conditions

Phenotypic variance ($\delta_{P}^{2}$) of each trait was partitioned into its components [replication ($\delta_{R}^{2}$), genotypic ($\delta_{G}^{2}$) and error ($\delta_{e}^{2}$)]. Higher genotypic variances (~2 and ~8.5-folds, respectively) were detected for germination rate and SPAD index in the flooded condition (Table 3). Higher genotypic variance of germination rate in flooded condition was further detected in race Durango/Jalisco (~32-fold) which is mostly due to a lack of genotypic diversity in non-flooded condition ($\delta_{G}^{2}=\;0.0251$). However, the genotypic variances of SPAD index were detected to be ~5 and ~9-folds higher in flooded condition for races Durango/Jalisco and Mesoamerica, respectively (Table 3). Genotypic variances of total, shoot, and root weight were higher (range from ~2 to ~10-folds) in non-flooded condition in the whole panel as well as each race separately (Table 3).

**Table 3:** Genotypic variance ($\delta_{G}^{2}$) of traits under non-flooded and flooded conditions.

	**Non-flooded**			**Flooded**		
**Trait**	**Whole panel**	**Durango/Jalisco**	**Mesoamerica**	**Whole panel**	**Durango/Jalisco**	**Mesoamerica**
Germination rate	0.7929	0.0251	1.9557	1.4386	0.8069	2.0884
Total weight	0.0351	0.0192	0.0094	0.0110	0.0047	0.0019
Shoot weight	0.0137	0.0087	0.0038	0.0059	0.0025	0.0010
Root weight	0.0058	0.0031	0.0018	0.0009	0.0006	0.0002
Hypocotyl length	1.3540	1.0723	0.8637	1.6351	1.3973	0.7867
SPAD index	8.9000	5.4253	6.7932	75.9761	26.3627	58.8868
Adventitious root^†^	–	–	–	0.3264	0.2553	0.1738

† *Adventitious root was observed only in flooded condition*.

### Relationship among traits

Regression analysis between greenhouse and field data indicates that strong significant regressions exists between the SPAD index measured in the greenhouse condition with survival rate and SPAD index measured in the field (Figure S4, *R*^2^ = 0.79 and *R*^2^ = 0.61, respectively).

**Figure 3:**
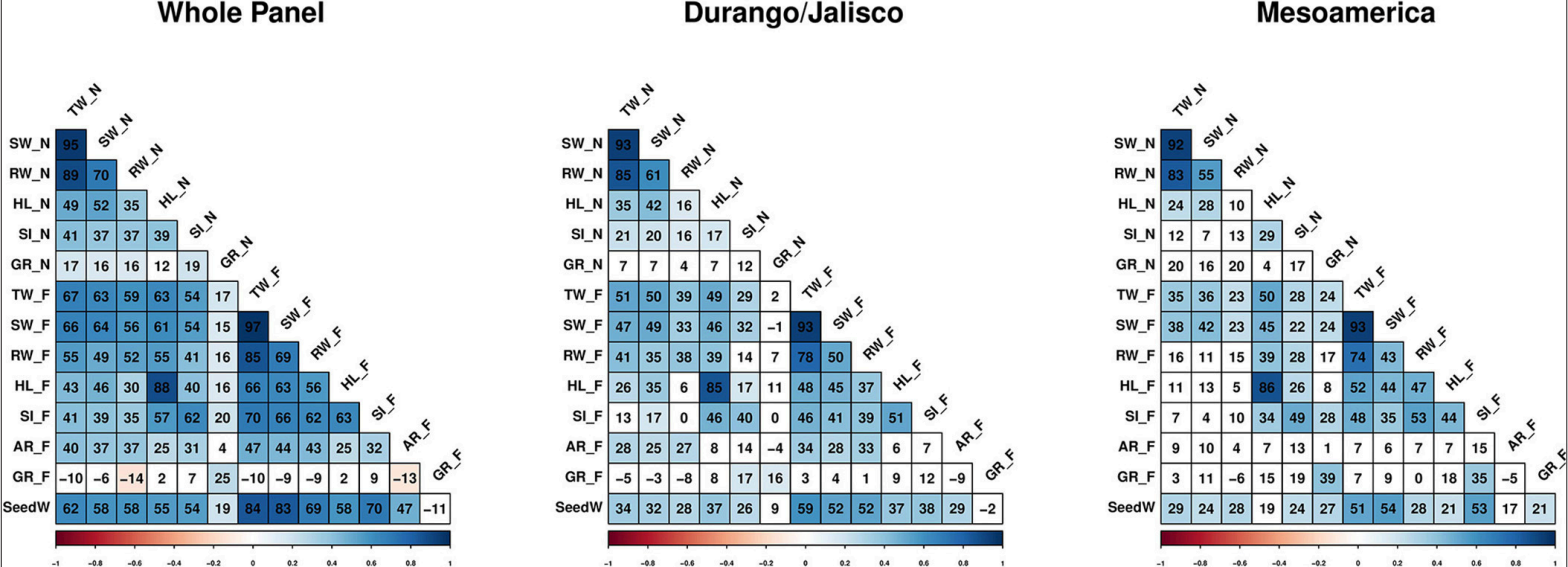
Correlation heatmaps among traits, within and across races. Values within each cell represents the Pearson correlation coefficient × 100. Non-significant correlations indicated by white cells. Traits evaluated in non-flooded and flooded conditions represented by _N and _F, respectively after the name of the trait. TW, Total weight; SW, shoot weight; RW, root weight; HL, hypocotyl length; SI, SPAD index; GR, germination rate; AR, Adventitious root formation and SeedW, seed weight.

Correlation coefficients among traits were measured at two different levels: (1) the whole panel consisting of all genotypes, and (2) within each race. When the whole population was considered, the correlation between root weight and SPAD index in flooding condition was significantly higher (*Z* = 3.9, *P* = 0) than the same correlation in non-flooded condition (Figure 3). This indicates that in flooded conditions, a strong positive correlation exists between root weight and chlorophyll content.

Moreover, the correlation between SPAD index and shoot weight was significantly higher in the flooding conditions in Durango/Jalisco (*Z* = 2.04, *P* = 0.04), Mesoamerica (*Z* = 2.05, *P* = 0.005), and the whole population (*Z* = 4.69, *P* = 0) compared with the non-flooded (Figure 3).

Even though the analysis of variance indicates that hypocotyl length was not affected by the treatment (reflected with *r* > 0.80 between HL_N and HL_F), the correlation study revealed that genotypes with longer hypocotyl tend to have ~ a 1.5 to 2-fold higher SPAD index values in stress conditions (Figure 3).

Seed weight data (Moghaddam et al., 2016) were included for the correlation analysis. Strong positive correlations were detected in the whole panel between seed weight and total weight (*r* = 0.84), shoot weight (*r* = 0.83), root weight (*r* = 0.69), and chlorophyll content (*r* = 0.70) under flooded condition.

### Detecting loci and candidate genes associated with traits in flooded condition

**Figure 4:**
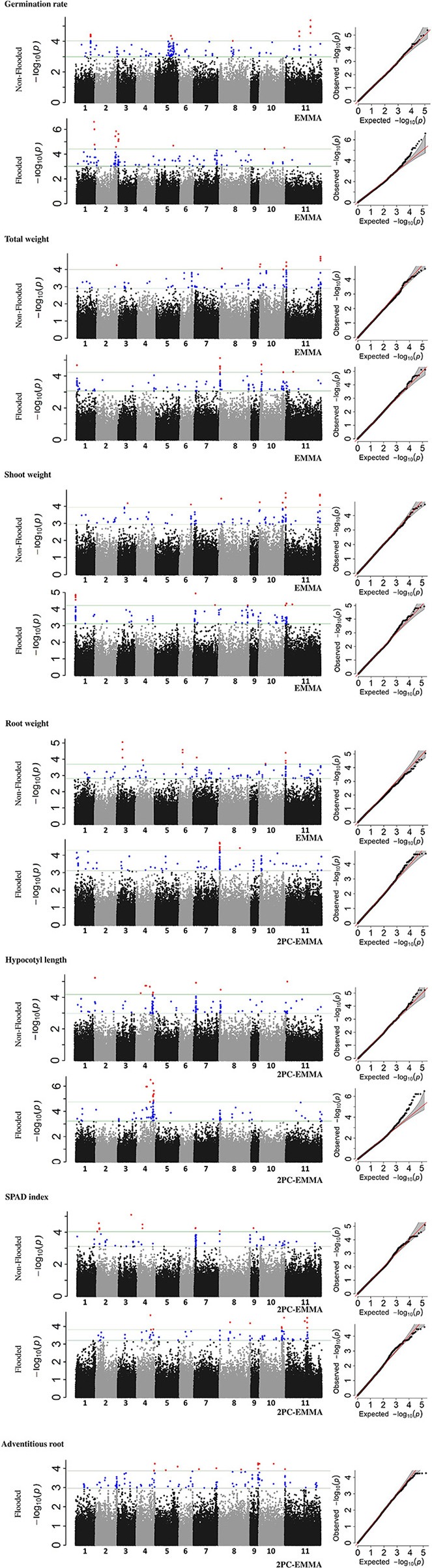
Manhattan and Q-Q plots, representing results of genome-wide association study (GWAS) in the whole MDP. Position of loci associated with seven traits in non-flooded and flooded conditions are represented. Blue circles in Manhattan plots represent loci with 0.1% < *P*-value < 1% and red circles represent loci with *P* < 0.1%.

Overall, 32 and 28 genomic regions were detected that were associated with traits in non-flooded and flooded conditions, respectively (Figure 4 and Table 4). Furthermore, a total of 28 genomic regions were associated with multiple traits under different treatment conditions (Table S2). A region was detected on Pv08/1.6 Mb that associates with five traits under flooding. This region was the most significant (*P* < 0.01) for root and total weight. At position Pv01/1.2 Mb, a major effect was observed for total and shoot weight in flooded condition. A major QTL hot spot associated with total weight, shoot weight, and root weight in the non-flooded condition is located at Pv11/1.5 Mb. Under this condition, a locus at Pv11/48.8 Mb is also associated with total weight and shoot weight.

**Table 4:** Major loci (*P* < 0.01) associated with traits in non-flooded and flooded conditions.

**Trait**	**Locus abbr**.	**Chr**	**Position**	**Class^†^**	**−log10 (*P*-value)**	***R^2^***
**GERMINATION RATE**						
Non-flooded	Pv01/32.6	Pv01	32,622,532	B	4.4	6.9
	Pv05/23.9	Pv05	23,955,828	B	4.3	9.6
	Pv08/28.9	Pv08	28,979,422	B	4.0	6.2
	Pv11/24.4	Pv11	24,454,851	B	4.6	7.3
	Pv11/35.6	Pv11	35,651,944	B	5.4	8.6
						28.3
Flooded	Pv01/42.4	Pv01	42,441,418	C	6.0	9.9
	Pv02/36.9	Pv02	36,954,028	C	5.4	10.9
	Pv02/41.0	Pv02	41,065,204	C	5.8	10.1
	Pv03/0.1	Pv03	100,011	C	5.6	9.3
						28.4
**TOTAL PLANT WEIGHT**						
Non-flooded	Pv08/6.8	Pv08	6,844,877	B	4.1	6.2
	Pv10/0.7	Pv10	710,077	B	4.1	7.0
	Pv10/35.3	Pv10	35,332,331	*A*^‡^	4.0	7.0
	Pv11/1.6	Pv11	1,592,653	*A*	4.4	8.1
	Pv11/48.8	Pv11	48,838,017	B	4.7	7.6
						24.0
Flooded	Pv01/1.1	Pv01	1,117,480	C	4.7	7.4
	Pv08/1.6	Pv08	1,654,593	C	5.1	8.2
	Pv10/2.8	Pv10	2,862,536	C	4.7	7.7
	Pv10/33.9	Pv10	33,949,345	C	4.2	6.6
						21.5
**SHOOT WEIGHT**						
Non-flooded	Pv03/16.7	Pv03	16,175,327	B	3.9	6.0
	Pv06/21.7	Pv06	21,782,651	B	4.1	6.7
	Pv08/6.8	Pv08	6,808,594	B	4.4	6.9
	Pv10/1.1	Pv10	1,175,810	B	4.2	7.7
	Pv10/35.3	Pv10	35,300,837	B	4.2	10.1
	Pv11/1.5	Pv11	1,538,514	*A*	4.5	8.1
	Pv11/48.8	Pv11	48,838,017	B	4.7	7.4
						30.5
Flooded	Pv01/1.2	Pv01	1,266,350	C	4.8	3.1
	Pv07/7.7	Pv07	7,759,867	*A*	4.9	2.0
	Pv08/55.5	Pv08	55,558,872	C	4.2	4.0
	Pv11/1.2	Pv11	1,296,339	*A*	4.2	4.0
	Pv11/4.3	Pv11	4,316,178	C	4.3	0.6
						11.0
**ROOT WEIGHT**						
Non-flooded	Pv03/14.1	Pv03	14,169,068	B	5.0	8.0
	Pv06/4.2	Pv06	4,267,347	B	4.6	7.2
	Pv07/9.9	Pv07	9,970,668	B	4.1	6.5
	Pv10/10.6	Pv10	10,675,818	B	3.7	6.1
	Pv11/1.5	Pv11	1,592,653	B	4.4	8.1
						29.0
Flooded	Pv08/1.6	Pv08	1,658,873	C	4.7	10.3
**HYPOCOTYL LENGTH**						
Non-flooded	Pv02/0.7	Pv02	723,951	*A*	5.2	8.4
	Pv04/42.2	Pv04	42,259,632	A	4.3	7.4
	Pv04/31.4	Pv04	31,493,716	*A*	4.7	8.1
	Pv08/4.7	Pv08	4,728,567	*A*	4.5	7.1
	Pv07/7.3	Pv07	7,389,394	B	4.9	7.9
	Pv11/3.0	Pv11	3,032,851	B	5.0	8.0
						34.1
Flooded	Pv04/3.1	Pv04	3,149,3716	*A*	6.5	11.1
	Pv04/42.2	Pv04	42,259,632	A	4.3	9.6
	Pv04/24.9	Pv04	24,941,687	*A*	5.9	10.0
						14.2
**SPAD INDEX**						
Non-flooded	Pv02/7.3	Pv02	7,391,330	*A*	4.2	3.1
	Pv04/11.9	Pv04	11,961,696	*A*	4.5	3.3
	Pv07/3.5	Pv07	3,545,443	B	4.2	4.1
	Pv08/0.4	Pv08	450,713	B	4.1	0.9
						6.4
Flooded	Pv04/41.2	Pv04	41,273,376	C	3.8	6.0
	Pv08/23.6	Pv08	23,662,637	C	4.2	6.6
	Pv10/30.2	Pv10	30,225,246	C	4.0	6.3
	Pv10/36.7	Pv10	36,735,566	C	4.5	7.0
	Pv11/32.2	Pv11	32,258,703	C	3.9	6.1
						24.4
**ADVENTITIOUS ROOTS^§^**						
Flooded	Pv04/43.9	Pv04	43,939,471	C	3.9	6.2
	Pv05/38.5	Pv05	38,510,730	C	4.1	6.4
	Pv07/45.1	Pv07	45,183,105	C	4.0	6.4
	Pv09/35.3	Pv09	35,326,516	C	4.3	8.8
	Pv10/0.8	Pv10	837,086	C	4.2	6.7
	Pv11/0.7	Pv11	770,275	C	3.9	6.5
						29.0

† *Classification was based on detection of QTL in each treatment. (A) QTL were detected in both treatments (non-flooded vs. flooded). (B) QTL just detected in the non-flooded condition and (C) the third group was detected in flooding condition*.

‡ *Class A QTL which are highlighted by italic font indicate that the corresponding locus in another treatment had the 0.01 < P-value < 0.1*.

§ *Adventitious root was observed only in flooded condition*.

To identify race-specific loci that are associated with stress, GWAS was performed on each race, separately (Figure S5, Table S3). Several race-specific loci were identified including two regions on Pv03/32.0 and Pv05/39.4 that were specifically associated with shoot weight in Durango/Jalisco subpopulation under flooding condition. Furthermore, a strong peak on Pv10/15.2 was associated with the SPAD index in race Mesoamerica. This peak, which was not detected in the whole-panel GWAS, controls for 19.7% of chlorophyll content variation in this race.

### Favorable allele frequency within races and market classes

The favorable allele frequencies (alleles with increasing effect on the trait values) for each significant GWAS peak under flooded conditions were calculated for each race and market class (Table 5). Black and small red beans possess the highest amount of favorable alleles for germination rate (0.25 and 0.19, respectively). However, pinto and great northern beans had the highest favorable alleles associated with total and shoot weight values. Interestingly, the favorable allele for root weight was fixed (*f* = 1.00) in pinto, great northern and navy, and it was the minor allele (*f* = 0.32) in the pink market class. Several cases were detected in which the alternative alleles had higher frequencies between races. Modification in fixation direction among races was detected for Pv10/2.8 and Pv10/33.9 controlling total plant weight, Pv08/55.5 associated with shoot weight, Pv10/30.2 for SPAD index and Pv04/43.9 and Pv09/35.3 for adventitious root (Table 5).

**Table 5:** Favored allelic effect and their frequencies within and across races and market classes under flooding conditions.

	**Nucleotide^†^**	**Allelic effect**	**Whole panel^‡^**	**Race**		**Market class**					
				**Durango/Jalisco**	**Mesoamerica**	**Pinto**	**GN**	**Pink**	**Small red**	**Black**	**Navy**
**GERMINATION RATE**											
Pv01/42.4	C/A	0.53	0.22	0.19	0.26	0.23	0.14	0.09	0.15	0.36	0.24
Pv02/36.9	A/T	0.75	0.10	0.01	0.24	0.00	0.00	0.14	0.27	0.26	0.04
Pv02/41.0	C/T	0.95	0.05	0.02	0.1	0.00	0.00	0.14	0.19	0.07	0.04
Pv03/0.1	A/G	0.04	0.11	0.03	0.23	0.03	0.00	0.09	0.15	0.33	0.09
Average		0.57	0.12	0.06	0.21	0.06	0.03	0.11	0.19	0.25	0.10
**TOTAL WEIGHT**											
Pv01/1.1	T/A	0.03	0.29	0.47	0.02	0.66	0.39	0.09	0.12	0.00	0.04
Pv08/1.6	C/T	0.05	0.92	0.87	1.00	1.00	1.00	0.32	0.81	0.98	1.00
Pv10/2.8	C/T	0.05	0.58	0.92	0.05	0.92	1.00	0.77	0.62	0.10	0.09
Pv10/33.9	T/C	0.04	0.60	0.92	0.08	0.93	0.96	0.77	0.62	0.14	0.11
Average		0.04	0.59	0.79	0.29	0.88	0.84	0.49	0.54	0.30	0.31
**SHOOT WEIGHT**											
Pv01/1.2	C/T	0.02	0.34	0.54	0.03	0.83	0.32	0.09	0.08	0.02	0.04
Pv07/7.7	G/A	0.03	0.25	0.40	0.02	0.60	0.04	0.14	0.19	0.02	0.04
Pv08/55.5	G/T	0.02	0.54	0.78	0.16	0.77	1.00	0.64	0.42	0.05	0.33
Pv11/1.2	T/C	0.01	0.29	0.41	0.11	0.44	0.54	0.27	0.23	0.10	0.13
Pv11/4.3	T/C	0.02	0.32	0.53	0.01	0.61	0.39	0.27	0.42	0.02	0.02
Average		0.02	0.35	0.53	0.07	0.65	0.46	0.28	0.27	0.04	0.11
**ROOT WEIGHT**											
Pv08/1.6	A/T	0.02	0.92	0.87	1.00	1.00	1.00	0.32	0.81	0.98	1.00
**HYPOCOTYL LENGTH**											
Pv04/3.1	G/A	0.68	0.90	0.83	1.00	0.84	1.00	0.64	0.85	1.00	1.00
Pv04/42.2	C/T	0.53	0.84	0.74	0.99	0.82	0.71	0.55	0.73	0.98	1.00
Pv04/24.9	G/A	0.65	0.90	0.83	1.00	0.83	1.00	0.64	0.88	1.00	1.00
Average		0.62	0.88	0.8	0.99	0.83	0.90	0.61	0.82	0.99	1.00
**SPAD INDEX**											
Pv04/41.2	G/A	3.10	0.90	0.84	1.00	0.85	0.96	0.68	0.85	1.00	1.00
Pv08/23.6	T/G	3.58	0.93	0.89	1.00	0.92	1.00	0.68	0.88	1.00	1.00
Pv10/30.2	G/T	5.04	0.63	0.97	0.08	1.00	1.00	0.82	0.62	0.14	0.11
Pv10/36.7	C/T	2.69	0.77	0.97	0.45	1.00	1.00	0.86	0.73	0.57	0.41
Pv11/32.2	C/T	3.88	0.93	0.92	0.95	0.95	1.00	0.68	0.96	0.93	0.96
Average		3.66	0.83	0.92	0.69	0.94	0.99	0.74	0.81	0.73	0.69
**ADVENTITIOUS RATE**											
Pv04/43.9	G/A	0.21	0.38	0.60	0.05	0.78	0.43	0.27	0.12	0.12	0.07
Pv05/38.5	T/C	0.26	0.10	0.12	0.08	0.14	0.14	0.00	0.19	0.05	0.02
Pv07/45.1	G/A	0.19	0.62	0.71	0.46	0.72	0.75	0.73	0.46	0.26	0.67
Pv09/35.3	T/G	0.24	0.51	0.77	0.10	0.83	0.61	0.82	0.50	0.07	0.11
Pv10/0.8	G/C	0.29	0.10	0.14	0.03	0.22	0.04	0.05	0.04	0.02	0.04
Pv11/0.7	A/T	0.21	0.75	0.89	0.53	0.94	0.93	0.64	0.73	0.64	0.48
Average		0.23	0.41	0.54	0.21	0.60	0.48	0.42	0.34	0.19	0.23

† *The nucleotide with the positive effect (favorable allele) is represented first*.

‡ *The favorable allele frequency in the whole population including both races*.

### Synteny analysis with soybean

**Figure 5:**
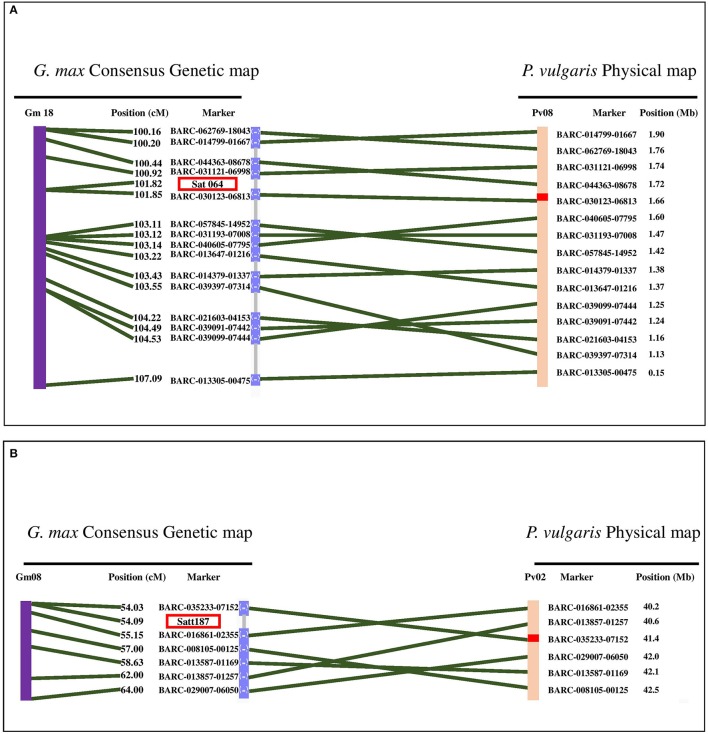
Synteny analysis between two QTL reported in soybean and the significant loci, detected in our study. **(A)** Position of marker Sat_064 which is associated with flooding tolerance in soybean is in synteny with Pv08/1.6 Mb which is associated with several traits in flooded condition in dry bean. **(B)** Satt187 is the marker associated with germination rate of soybean seeds under flooding condition. This region is in synteny with Pv02/ 41.1 Mb which is associated with germination rate under flooding condition. Green lines represent the synteny of homologous regions between soybean and dry bean chromosomes.

Two flooding-related QTL in soybean were detected that are syntenic with flooding-associated loci in our analysis (Figure 5). The first soybean QTL was located on Gm18 in an interval between 55.6 and 58.0 Mb. This region is syntenic with the Pv08/0.14-5.69 Mb peak in this analysis. Micro-synteny analysis revealed that Sat_064, the closest flooding-associated marker in soybean (VanToai et al., 2001; Valliyodan et al., 2016), is located between 1.6 and 1.7 Mb of Pv08 (Figure 5A). In our experiment, this region is detected to be associated with multiple traits but most significantly with root weight under flooding (Table S2).

The second case of phenotypic synteny was a QTL that controls soybean seed germination under excess water condition. This QTL is located at Gm08/8.9-12.1 Mb. The closest soybean marker to the QTL is Satt187 (Sayama et al., 2009) which is homologous to Pv02/40.0-41.95 Mb. Micro-synteny analysis revealed that this marker is located between Pv02/40.2-41.4 Mb (Figure 5B), a position associated with germination rate under flooding in common bean.

## Discussion

Flooding is a major abiotic stress affecting crop production worldwide. Legumes, including common bean, are some of the most sensitive crop species to flooding condition, especially at early growth stages. To understand the mechanism of flooding tolerance and its genetic components in the Middle-American gene pool of dry bean, we analyzed the effect of this stress at the germination and seedling stages under greenhouse conditions, given the fact that a large field flooding experiment is logistically difficult and inaccurate. Nonetheless, a subset of lines was further evaluated in the field to assess the relationship between field and greenhouse results. Strong correlation (Figure S4) between the field and greenhouse data indicates the reliability of a greenhouse screening at early developmental stages for flooding tolerance. This is especially important for breeding programs who need to evaluate numerous genotypes. Significant genotype × treatment interactions were detected for five traits evaluated at the two stages (Table 1). The interaction component indicates differential response of the population under flooding and control conditions. The detection of a significant interaction for these traits provides evidence of the existence of genotypic polymorphisms among MDP lines which contribute to a differential response to flooding.

### Vestiges of eco-geographic variations in phenotypic response to flooding

The Middle-American gene pool consists of races Durango, Jalisco, and Mesoamerica. It was proposed that these races evolved and adapted to different eco-geographic niches in the region (Singh et al., 1991). Race Mesoamerica is mainly distributed throughout tropical lowlands of Mexico and Central America, while race Durango evolved in the semiarid highlands of Mexico, and race Jalisco is indigenous to the humid highlands of Mexico (Singh et al., 1991). Morpho-physiological variation, especially for seed size and pigmentation, distinguishes these races enable each race to survive specific biotic and abiotic constrains. That variation was also observed by a differential response to flooding conditions.

Seedling growth and germination rate distinguished the two races in the first two principal components, and except for hypocotyl length and germination rate, response to flooding (as indicated by the flooding index) was affected collectively by all of the other traits when the factor analysis was performed. This indicates that under flooding condition, the phenotypic traits evaluated here are diagnostic for the two races (Figure 2). Chlorophyll content distinguished the two races (Figure 1 and Table 2). That is consistent with previous studies where Mexican landraces of race Durango/Jalisco had higher chlorophyll content than race Mesoamerica Guatemalan landraces (Gonzalez et al., 1995). These morpho-physiological race differences may reflect adaptation mechanisms to different geographic and environmental regions and conditions.

The small-seeded black and small red beans possessed the highest rates of germination under flooding stress (Figure 1). Interestingly, it has been shown that soybean seed tolerance to flooding is a function of seed size and pigmentation (Hou and Thseng, 1992; Sayama et al., 2009). This similarity between these dry bean market classes and soybean may indicate an evolutionary conserved mechanism in these species.

In general, the fixation rates of significant GWAS peaks trended to 0 or 1 for race Durango/Jalisco and Mesoamerica (Table 5). For some loci, each race had an equally high fixation rates but for alternative alleles. As an example, Pv10/30.2 Mb, which is highly associated with the SPAD index, is equally fixed for different alleles in both races in flooding condition. This suggests that each allele conditions fitness to a different environmental condition for each race during either its domestication or breeding history. Several other cases suggest antagonistic pleiotropy exists between races Durango/Jalisco and Mesoamerica (Table 5). Although the fixation for the favorable allele of Pv08/1.6 Mb, which controls root weight, was *f* > 0.80 among pinto, great northern, small red, black and navy beans, the alternative allele was more frequent in pink beans (Table 5). This might reflect a historical selection event unique to pink-seeded beans. Although the current study highlights the intrinsic differences among races, the MDP consists of modern varieties with an extensive history of intra- and inter-racial hybridizations. Thus, separate studies on wild and landrace populations are needed to better understand the process of local adaptation and allelic selection in *Phaseolus vulgaris* populations.

### Underlying genetic factors associated with flooding tolerance

Except hypocotyl length, the majority of genomic loci associated with the traits were unique for each condition (flooded vs. non-flooded). This indicates the phenotypic plasticity of each trait and inducibility of underlying genes by environmental parameters. Based on this experiment, flooding tolerance seems to be a polygenic trait controlled by several loci, as observed with other studies (Osman et al., 2013). Higher genotypic variances were detected in flooded stress only for germination rate and SPAD index (Table 3). On the contrary, genotypic variances of total weight, shoot and root weight were higher in the non-flooded condition. It was proposed that morphometric traits possess higher heritability in favorable environments (Visscher et al., 2008), presumably due to the possibility to reach their genetic potential in non-stress condition (Hoffmann and Merilä, 1999). However, traits that are more closely associated with the fitness have a higher heritability under stress condition (Visscher et al., 2008). This might be due to greater selection against low-fitness alleles under common favorable environments compared with less common unfavorable environments (Hoffmann and Merilä, 1999).

Two genes located in the GWAS peaks with the highest *R*^2^ for germination rate under flooding are possible candidates. Alternative oxidase 2 (*AOX2*, Table S4) maps near the Pv02/36.9 Mb peak. Alternative oxidase pathway is crucial in metabolic homeostasis, particularly under biotic and abiotic stress (Vanlerberghe, 2013), and the gene is important during the early germination stages of *Arabidopsis* (Saisho et al., 2001). *Phvul.002G243600* which encodes a trehalose-6-phosphate synthase (Table S4) is located ~19.8 Kb downstream of the Pv02/41 Mb peak for germination rate under flooding. Trehalose-6-phosphate is a sugar signaling molecule and it is crucial in sugar perception and germination under anaerobic condition (Kretzschmar et al., 2015; Loreti et al., 2016). Furthermore, this gene interacts with SnRK1 (Tsai and Gazzarrini, 2014), another gene involved in sugar perception under hypoxia (Baena-González et al., 2007).

Total weight, shoot weight and root weight at the seedling stage are correlated with seed weight (Figure 3). Genomic regions associated with seed weight were detected on Pv10 (Moghaddam et al., 2016). This region was also associated with total and shoot weight in flooded condition (Figure 4). Thus, the same gene(s) on Pv10 might be pleiotropic for these traits. However, regions on other chromosomes were detected that are associated with seedling weight that are independent of seed weight. For instance, a QTL located at Pv08/55.6 Mb was associated with shoot weight in flooding condition and resides in the vicinity of sucrose synthase (*SUS*). In hypoxic conditions, some species shift the sucrose degradation from invertase to SUS to reduce the ATP consumption (Bailey-Serres et al., 2012a). Chloroplastic choline monooxygenase (*Phvul.010G082000*) is located within 122 kb of one of the GWAS peaks (Pv10/30.2 Mb) associated with SPAD index. This gene is involved in biosynthesis of glycine betaine which improves the tolerance level of plants to various abiotic stresses (Giri, 2011). It was shown that the accumulation of glycine betaine can preserve the chlorophyll content of tobacco (*Nicotiana tabacum* L.) plants under salt stress (Zhang et al., 2008).

Roots are the most adversely affected plant structure by flooding (Rocha et al., 2010; Sauter, 2013). Prolonged hypoxia/anoxia results in perturbations in root hydraulic conductance and mineral uptake which causes stomatal closure, wilting, and chlorosis in leaves (Samet and Sinclair, 1980; Bailey-Serres and Voesenek, 2010; Colmer and Greenway, 2011). A QTL under flooding conditions was identified at Pv08/1.6 Mb for root weight, total weight, shoot weight, hypocotyl length and SPAD index suggesting a pleiotropic effect of a locus at this location. This region is syntenic with a QTL in soybean associated with growth and grain yield under flooding that explained 22–33% of grain yield variation in two soybean segregating populations (VanToai et al., 2001; Cornelious et al., 2006). The discovery of the syntenic QTL (Figure 5) suggest a conserved evolutionary mechanism between dry bean and soybean that predates the separation of these species ~19.2 MYA. The marker with the lowest *P*-value on Pv08/1.6 Mb is located ~53 Kb upstream of *Phvul.008G019600* which is a homolog of *RAP2.6L* (Table S4), a gene that belongs to the ERF gene family (Nakano et al., 2006) and is involved in *Arabidopsis* tolerance to flooding stress (Liu et al., 2012). RAP2.6L belongs to the AP2/ERF transcription factor family which regulates several downstream genes involved in different pathways, which would explain the pleiotropic effects observed for this region. *RAP2.6L* is an abscisic acid (ABA)-dependent gene and improves flooding tolerance by ameliorating hydraulic and oxidative status of the stressed plant. MYB, NAC, and WRKY cis-elements were observed in the 5′ region of this gene (Table S4). MYB and WRKY motifs were also reported in the promoter of *Arabidopsis RAP2.6L* (Liu et al., 2012) suggesting a conserved deep evolutionary regulatory mechanism for this gene. The *RAP2.6L* gene controls stomatal conductance in flooding stress (Liu et al., 2012). Stomatal conductance control also has shown to be beneficial in drought tolerance in common bean, especially within the Durango race (Acosta-Díaz et al., 2009; Beebe et al., 2013). However, the potential role of RAP2.6L in controlling stomatal conductance in drought and flooding tolerance mechanisms in dry bean remains to be clarified in future studies.

The major locus associated with adventitious root formation under flooding was located on Pv09/9.4 Kb from an Ankyrin repeat-containing protein (*Phvul.009G240400)*. A member of the ankyrin-protein kinase family was shown to be involved in the perception of abiotic stress and the induction of adventitious roots production in *Arabidopsis* (Chinchilla et al., 2008). Adventitious roots play a significant role in an escape strategy by providing oxygen, water, and minerals to the plant (Voesenek and Bailey-Serres, 2013; Steffens and Rasmussen, 2016).

## Conclusion

If climate change persists, more frequent incidences of excess water in some regions will lead to food insecurity. While dry bean is susceptible to excess water, tolerance can be improved by adopting an efficient breeding strategy based on a comprehensive understanding of flooding tolerance mechanisms in common bean. The results indicate that, in general, race Durango/Jalisco was the most tolerant at the seedling stage, whereas pigmented small seeded genotypes were the most tolerant at the germination stage. From an evolutionary point of view, these differences can be interpreted as different adaptation trajectories among various races of common bean. Several loci and candidate genes were associated with tolerance to flooding stress. These loci can be targets for marker-assisted selection. Although this study provides information about tolerance mechanisms within the Middle-American gene pool, further research is needed to elucidate the physiological pathways involved in flooding tolerance in the Andean gene pool. Screening wild beans and landraces as well as related species would be beneficial for identifying new sources of tolerance.

## Author contributions

AS, KW, SS, and RL performed the experiment; AS, SMM, DR, and SM contributed to analysis; AS, JO, and PM designed the experiment and wrote and edit the manuscript.

### Conflict of interest statement

The authors declare that the research was conducted in the absence of any commercial or financial relationships that could be construed as a potential conflict of interest.

## Supplementary material

Figure S1 Phylogenetic tree of MDP genotypes. Each taxa node color-coded based on the market class in which pinto, green; great northern, yellow; pink, pink; small red, red; navy, blue and black, black.

Figure S2 Frequency distribution of seven traits in non-flooded (above) and flooded condition (below).

Figure S3 Scatterplots of flooding indices. TW, Total weight; SW, shoot weight; RW, root weight; HL, hypocotyl length; SI, SPAD index; GR, germination rate; AR, Adventitious root formation.

Figure S4 Regression between field and greenhouse results based on a subset of 21 lines. **(A)** Regression between survival rate (field) and SPAD index (greenhouse) and **(B)** regression between SPAD indices measured in the field and greenhouse.

Figure S5 Manhattan and Q-Q plots, representing results of genome-wide association study (GWAS) in each race (Durango/Jalisco and Mesoamerica). Position of loci associated with seven traits in non-flooded and flooded conditions are represented. Blue circles in Manhattan plots represent loci with 0.1% < *P*-value <1% and red circles represent loci with *P* < 0.1%.

Table S1 Rotated component matrix of first two PCs in each condition.

Table S2 List of genomic regions controlling multiples traits in flooded or non-flooded conditions.

Table S3 List of race-specific peaks (*P* < 0.01) associated with traits in flooded condition.

Table S4 List of potential candidate genes, associated with major significant loci, detected in flooded stress.
