# Supplementary figures and images for: Genetic Architecture of Flooding Tolerance in the Dry Bean Middle-American Diversity Panel
Source: Front Plant Sci. 2017 Jul 6;8:1183. doi: 10.3389/fpls.2017.01183 (PMC5498472; doi:10.3389/fpls.2017.01183)

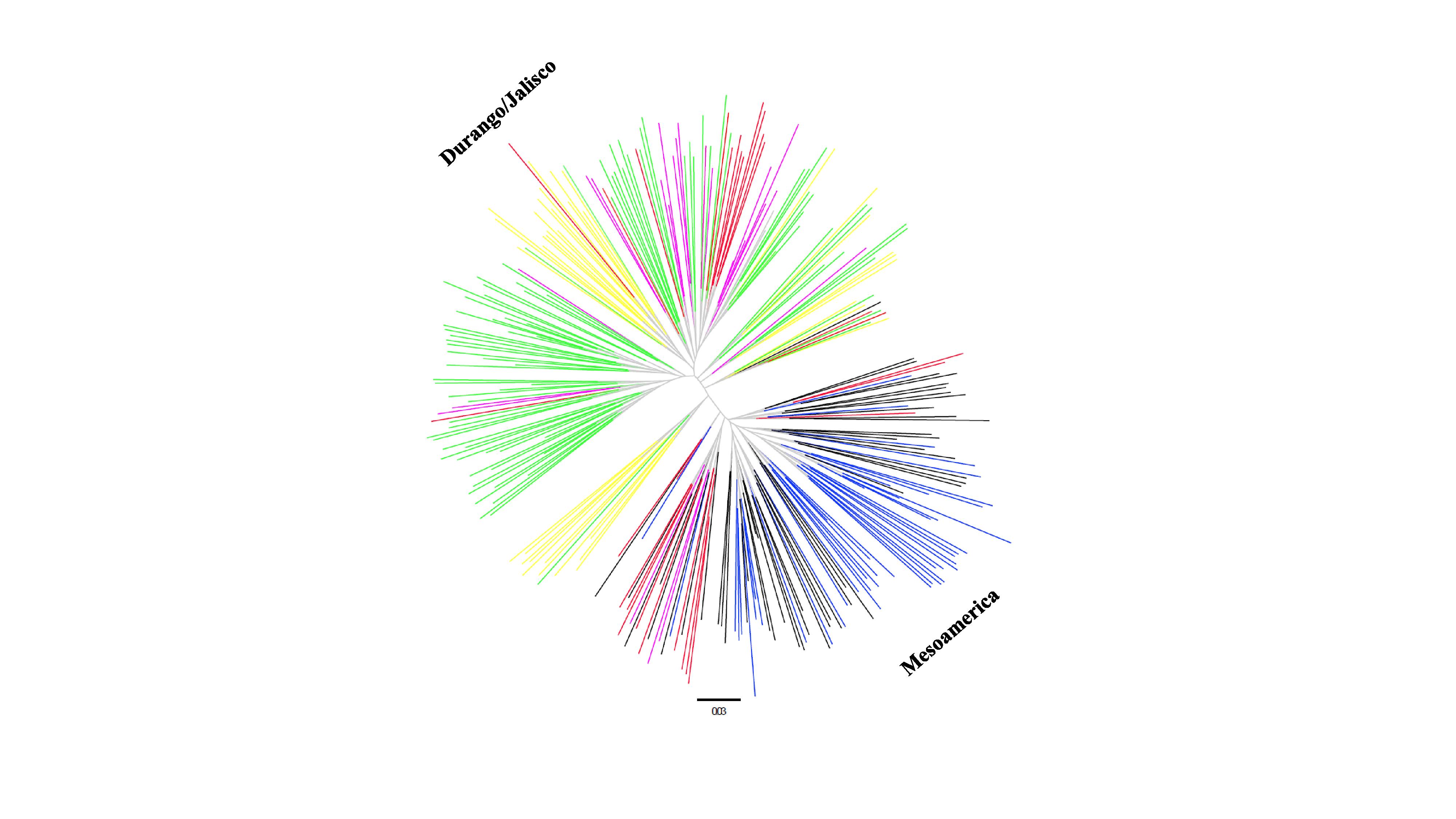

Supplement: Figure S1 — Phylogenetic tree of MDP genotypes. Each taxa node color-coded based on the market class in which pinto, green; great northern, yellow; pink, pink; small red, red; navy, blue and black, black. [file Image1.JPEG]

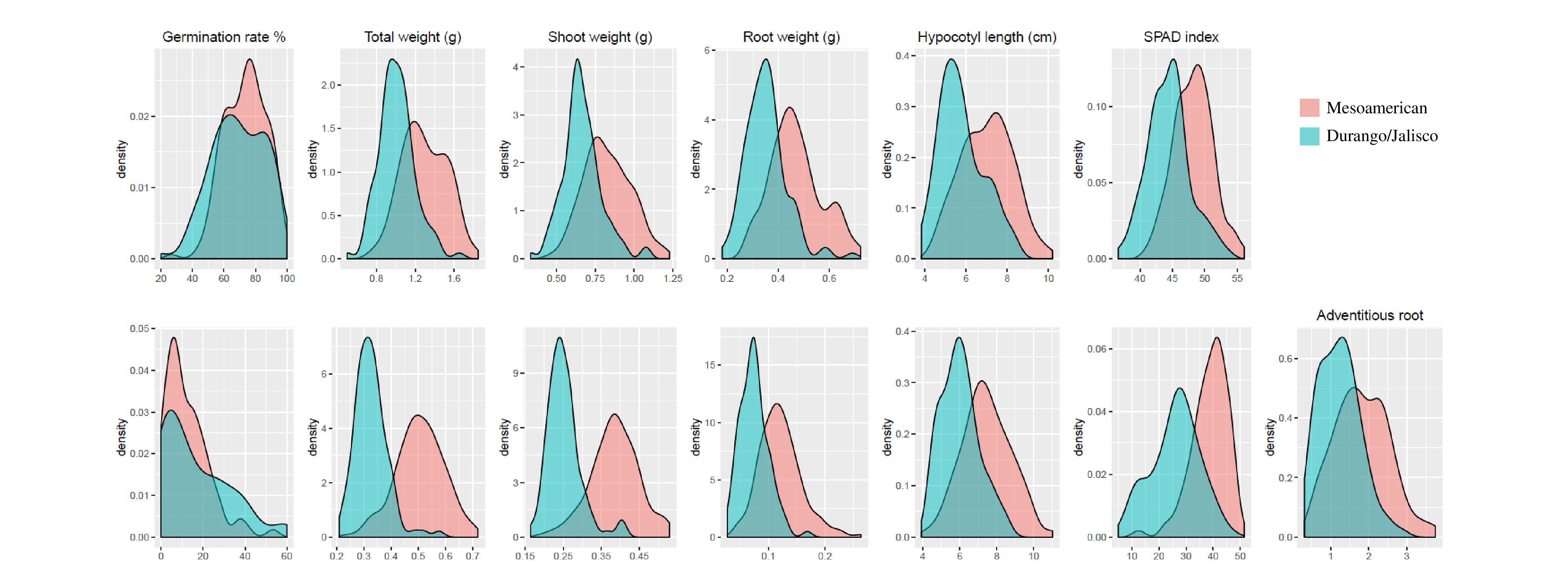

Supplement: Figure S2 — Frequency distribution of seven traits in non-flooded (above) and flooded condition (below). [file Image2.JPEG]

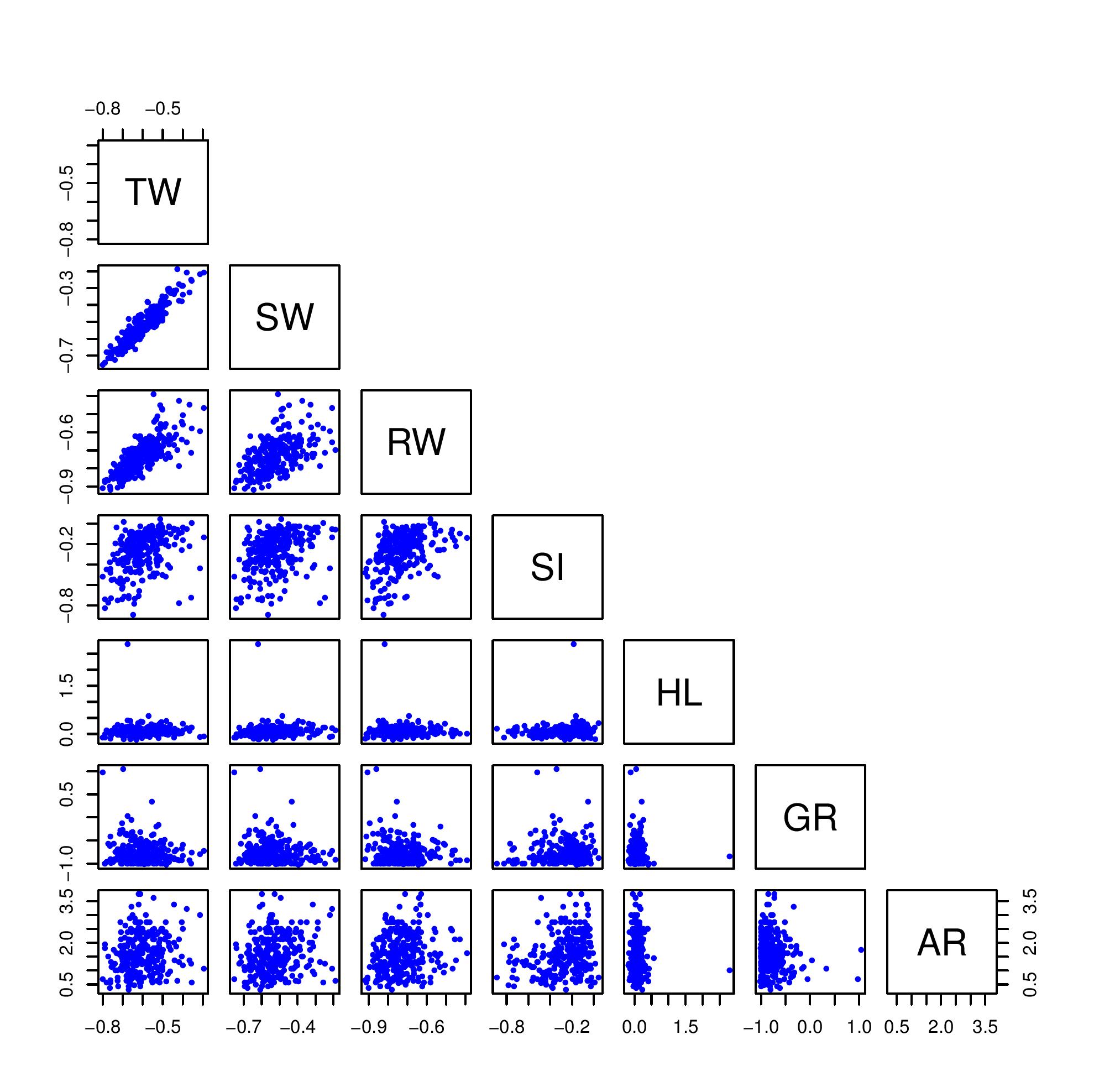

Supplement: Figure S3 — Scatterplots of flooding indices. TW, Total weight; SW, shoot weight; RW, root weight; HL, hypocotyl length; SI, SPAD index; GR, germination rate; AR, Adventitious root formation. [file Image3.JPEG]

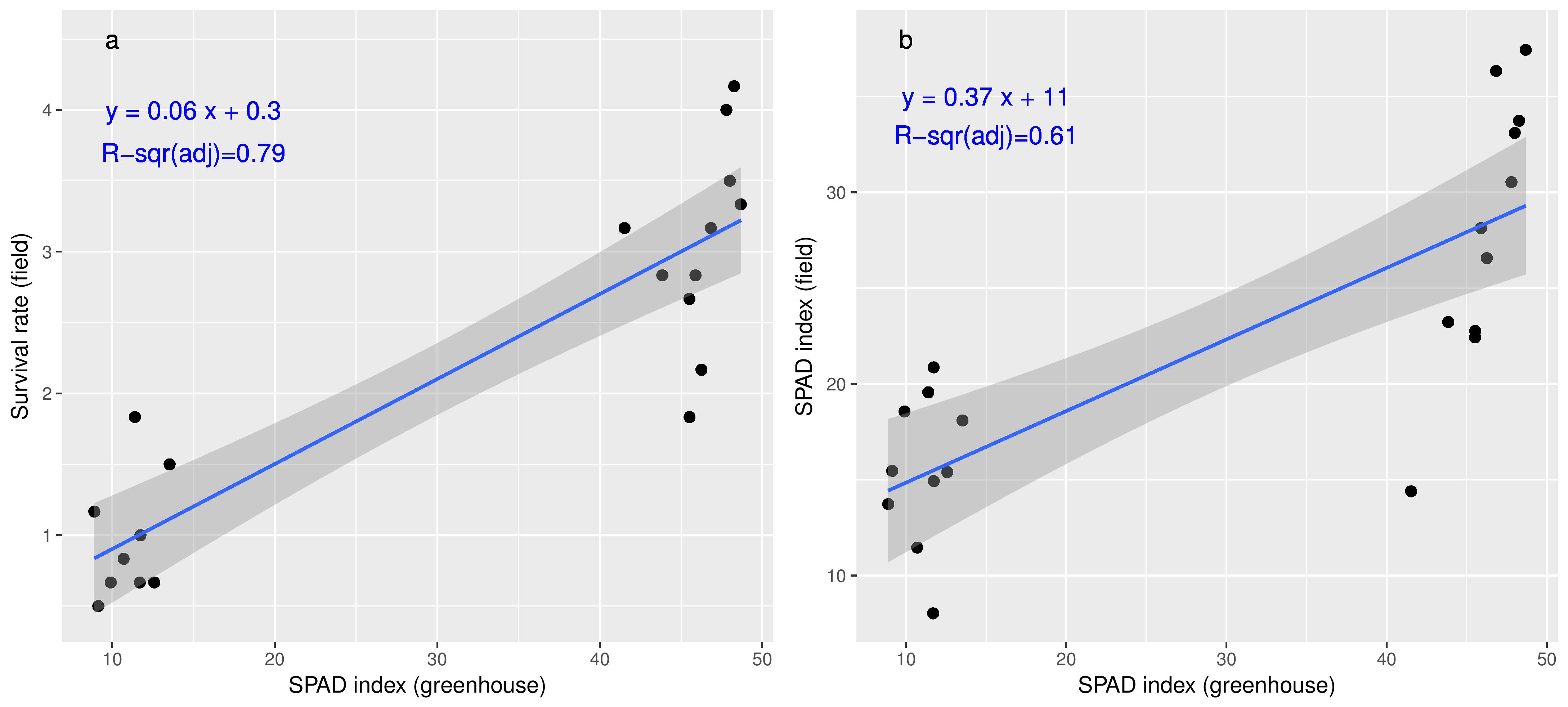

Supplement: Figure S4 — Regression between field and greenhouse results based on a subset of 21 lines. (A) Regression between survival rate (field) and SPAD index (greenhouse) and (B) regression between SPAD indices measured in the field and greenhouse. [file Image4.JPEG]
