# Supplementary material for: Genetic Architecture of Flooding Tolerance in the Dry Bean Middle-American Diversity Panel
Source: Front Plant Sci. 2017 Jul 6;8:1183. doi: 10.3389/fpls.2017.01183 (PMC5498472; doi:10.3389/fpls.2017.01183)
Supplement: Table S1 — Rotated component matrix of first two PCs in each condition. [file DataSheet2.docx]

| Table S1. Rotated component matrix of first two PCs in each condition | | | | | | | | | | | |  |
| --- | --- | --- | --- | --- | --- | --- | --- | --- | --- | --- | --- | --- |
|  | | Non-flooded | |  | Flooded | | |  | Flooding index | | |  |
|  |  | PC1 | PC2 |  | | PC1 | PC2 |  | | PC1 | PC2 | |
| Germination rate |  | -0.03 | 0.88 |  | | 0.09 | 0.91 |  | | -0.09 | -0.06 | |
| Total weight |  | 0.97 | 0.13 |  | | 0.94 | -0.19 |  | | 0.98 | 0.07 | |
| Shoot weight |  | 0.92 | 0.14 |  | | 0.90 | -0.17 |  | | 0.90 | 0.02 | |
| Root weight |  | 0.88 | 0.08 |  | | 0.84 | -0.20 |  | | 0.79 | 0.22 | |
| Hypocotyl length |  | 0.55 | 0.36 |  | | 0.80 | 0.07 |  | | 0.00 | -0.02 | |
| SPAD index |  | 0.40 | 0.56 |  | | 0.85 | 0.12 |  | | 0.42 | 0.60 | |
| Adventitious root † |  | - | - |  | | 0.46 | -0.49 |  | | 0.03 | 0.89 | |
| † Adventitious root was observed only in flooded condition. | | | | | | | | | | | | |

| Table S2. List of genomic regions controlling multiples traits in flooded or non-flooded conditions | | | | | | | | | | | | | | |
| --- | --- | --- | --- | --- | --- | --- | --- | --- | --- | --- | --- | --- | --- | --- |
|  | Non-flooded | | | | | |  | Flooded | | | | | | |
| Position | GR† | TW | SW | RW | HL | SI |  | GR | TW | SW | RW | HL | SI | AR |
| Pv01/1.2 |  |  |  |  |  |  |  |  | 4.7 | 4.8 |  |  |  |  |
| Pv01/8.5 |  |  |  |  |  |  |  |  | 3.5 |  | 3.9 |  |  |  |
| Pv01/13.9 |  |  |  |  | 3.9 |  |  |  |  |  |  | 4.2 |  |  |
| Pv02/0.7 |  |  |  |  | 5.2 |  |  |  |  |  |  | 4.1 |  |  |
| Pv02/22.9 |  |  | 3.1 | 3.2 |  |  |  |  |  | 3.3 |  |  |  |  |
| Pv03/14.1 |  | 3.2 |  | 5.0 |  |  |  |  |  |  |  |  |  |  |
| Pv04/11.9 |  |  |  |  |  | 4.5 |  |  |  |  |  |  | 3.7 |  |
| Pv04/31.5 |  |  |  |  | 4.7 |  |  |  |  |  |  | 6.5 |  |  |
| Pv04/42.1 |  |  |  |  | 4.3 |  |  |  | 4.0 | 3.6 |  | 4.3 |  |  |
| Pv05/38.5 |  |  |  |  |  |  |  |  |  |  |  |  | 3.2 | 4.1 |
| Pv06/21.8 |  | 3.7 | 4.1 |  |  |  |  |  |  |  |  |  |  |  |
| Pv07/3.6 |  |  | 3.6 |  |  | 4.1 |  |  |  |  |  |  |  |  |
| Pv07/4.2 |  |  |  |  |  |  |  |  | 3.1 | 3.4 |  |  |  |  |
| Pv07/7.2 |  | 3.0 | 3.1 |  | 4.9 |  |  |  |  |  |  |  |  |  |
| Pv08/1.6 |  |  |  |  |  |  |  |  | 5.1 | 3.9 | 4.7 | 4.1 | 3.8 |  |
| Pv08/4.7 |  |  |  |  | 4.5 |  |  |  |  |  |  | 3.4 |  |  |
| Pv08/55.5 |  | 3.3 |  | 3.0 |  |  |  |  | 3.5 | 4.2 |  |  |  |  |
| Pv08/58.6 |  |  |  |  |  | 3.6 |  |  |  |  |  |  | 4.2 |  |
| Pv10/0.7 |  | 4.1 |  | 3.2 |  |  |  |  |  |  |  |  |  | 4.2 |
| Pv10/2.8 |  |  |  |  |  |  |  |  | 4.7 | 3.8 |  |  |  |  |
| Pv10/4.1 |  |  |  |  |  |  |  |  |  |  |  |  | 3.4 | 3.4 |
| Pv10/10.7 |  | 3.9 | 3.3 | 3.7 |  |  |  |  |  |  |  |  |  |  |
| Pv10/33.8 |  |  | 3.2 |  |  |  |  |  | 4.2 | 3.5 |  |  |  |  |
| Pv10/35.3 |  | 4.0 | 4.2 |  |  |  |  |  | 3.2 |  |  |  |  |  |
| Pv11/1.5 |  | 4.4 | 4.5 | 4.4 |  |  |  |  |  |  |  |  |  |  |
| Pv11/46.8 |  | 3.4 | 3.6 |  |  |  |  |  |  |  |  |  |  |  |
| Pv11/48.8 |  | 4.7 | 4.7 |  |  |  |  |  |  |  |  |  |  |  |
| Pv11/50.2 |  | 3.1 |  |  |  |  |  |  |  |  |  | 3.2 |  |  |
| † Abbreviate name of the traits: TW: total weight, SW: shoot weight, RW: root weight, HL: hypocotyl length, AR: adventitious rate. | | | | | | | | | | | | | | |

| Table S3. List of race-specific peaks (P-value<0.01) associated with traits in flooded condition | | | | | | | | | | | | | | | | | | | | |
| --- | --- | --- | --- | --- | --- | --- | --- | --- | --- | --- | --- | --- | --- | --- | --- | --- | --- | --- | --- | --- |
| Trait |  | Race |  | Locus |  | -log10 (P-value) |  | R^2^ | |  | Nucleotide † |  | Allelic effect |  | Favorable allele frequency | | | | | |
| Germination rate |  |  |  |  |  |  |  |  |  |  |  |  |  |  | Pinto | GN | Pink | Red | Black | Navy |
|  |  | DJ |  | Pv06/14.7 |  | 4.0 |  |  | 11.4 |  | G/A |  | 0.78 |  | 0.02 | 0.07 | 0.09 | 0.38 | 0.07 | 0.07 |
|  |  |  |  | Pv07/25.8 |  | 4.4 |  |  | 14.6 |  | C/A |  | 0.62 |  | 0.09 | 0.00 | 0.18 | 0.08 | 0.31 | 0.20 |
|  |  |  |  | Pv07/36.6 |  | 4.3 |  |  | 13.9 |  | T/C |  | 0.54 |  | 0.10 | 0.18 | 0.09 | 0.19 | 0.36 | 0.26 |
|  |  |  |  |  |  |  |  | Total | 28.7 |  |  |  |  |  |  |  |  |  |  |  |
|  |  | MA |  | Pv02/41.0 |  | 4.0 |  |  | 17.7 |  | G/A |  | 1.07 |  | 0.16 | 0.00 | 0.14 | 0.15 | 0.07 | 0.04 |
|  |  |  |  | Pv06/26.5 |  | 4.7 |  |  | 20.9 |  | C/G |  | 1.38 |  | 0.14 | 0.32 | 0.09 | 0.08 | 0.07 | 0.00 |
|  |  |  |  |  |  |  |  | Total | 27.2 |  |  |  |  |  |  |  |  |  |  |  |
| Total weight |  |  |  |  |  |  |  |  |  |  |  |  |  |  |  |  |  |  |  |  |
|  |  | DJ |  | Pv01/1.1 |  | 3.7 |  |  | 8.6 |  | T/A |  | 0.02 |  | 0.66 | 0.39 | 0.09 | 0.12 | 0.00 | 0.04 |
|  |  |  |  | Pv05/39.4 |  | 3.8 |  |  | 9.7 |  | G/T |  | 0.03 |  | 0.70 | 0.61 | 0.36 | 0.50 | 0.60 | 0.41 |
|  |  |  |  | Pv08/1.6 |  | 3.9 |  |  | 10.1 |  | C/T |  | 0.04 |  | 1.00 | 1.00 | 0.32 | 0.81 | 0.98 | 1.00 |
|  |  |  |  | Pv10/33.9 |  | 3.8 |  |  | 10.2 |  | A/G |  | 0.05 |  | 0.93 | 0.96 | 0.77 | 0.62 | 0.14 | 0.11 |
|  |  |  |  |  |  |  |  | Total | 27.9 |  |  |  |  |  |  |  |  |  |  |  |
| Shoot weight |  |  |  |  |  |  |  |  |  |  |  |  |  |  |  |  |  |  |  |  |
|  |  | DJ |  | Pv01/1.3 |  | 4.2 |  |  | 10.8 |  | C/T |  | 0.02 |  | 0.66 | 0.39 | 0.09 | 0.12 | 0.00 | 0.04 |
|  |  |  |  | Pv03/32.0 |  | 4.3 |  |  | 11.7 |  | A/G |  | 0.03 |  | 0.90 | 0.82 | 0.73 | 0.42 | 0.07 | 0.13 |
|  |  |  |  | Pv05/39.4 |  | 4.0 |  |  | 10.6 |  | G/T |  | 0.02 |  | 0.70 | 0.61 | 0.36 | 0.50 | 0.60 | 0.41 |
|  |  |  |  | Pv011/5.0 |  | 4.2 |  |  | 10.9 |  | T/C |  | 0.02 |  | 0.67 | 0.32 | 0.77 | 0.46 | 0.07 | 0.09 |
|  |  |  |  |  |  |  |  | Total | 33.2 |  |  |  |  |  |  |  |  |  |  |  |
| Root weight |  |  |  |  |  |  |  |  |  |  |  |  |  |  |  |  |  |  |  |  |
|  |  | DJ |  | Pv01/26.7 |  | 4.9 |  |  | 13.2 |  | T/C |  | 0.02 |  | 0.08 | 0.11 | 0.09 | 0.04 | 0.07 | 0.13 |
|  |  |  |  | Pv08/1.6 |  | 3.7 |  |  | 10.3 |  | C/T |  | 0.02 |  | 1.00 | 1.00 | 0.32 | 0.81 | 0.98 | 1.00 |
|  |  |  |  |  |  |  |  | Total | 21.3 |  |  |  |  |  |  |  |  |  |  |  |
|  |  | MA |  | Pv10/19.6 |  | 4.3 |  |  | 19.6 |  | C/T |  | 0.02 |  | 0.87 | 0.82 | 0.73 | 0.35 | 0.17 | 0.07 |
|  |  |  |  | Pv11/47.1 |  | 3.7 |  |  | 15.0 |  | A/G |  | 0.01 |  | 0.47 | 0.39 | 0.59 | 0.35 | 0.29 | 0.28 |
|  |  |  |  |  |  |  |  | Total | 28.3 |  |  |  |  |  |  |  |  |  |  |  |
| Hypocotyl length |  |  |  |  |  |  |  |  |  |  |  |  |  |  |  |  |  |  |  |  |
|  |  | DJ |  | Pv04/39.4 |  | 6.3 |  |  | 17.5 |  | C/G |  | 0.62 |  | 0.79 | 0.61 | 0.45 | 0.73 | 0.98 | 1.00 |
|  |  | MA |  | Pv04/26.7 |  | 3.9 |  |  | 16.7 |  | C/T |  | 0.35 |  | 0.76 | 0.68 | 0.86 | 0.73 | 0.67 | 0.59 |
|  |  |  |  | Pv08/10.8 |  | 3.9 |  |  | 16.2 |  | G/A |  | 0.49 |  | 0.94 | 1.00 | 0.82 | 0.65 | 0.21 | 0.26 |
|  |  |  |  | Pv08/16.2 |  | 4.3 |  |  | 18.5 |  | T/C |  | 0.53 |  | 0.92 | 0.96 | 0.82 | 0.69 | 0.26 | 0.26 |
|  |  |  |  | Pv10/3.3 |  | 3.6 |  |  | 14.7 |  | T/C |  | 0.79 |  | 0.91 | 0.89 | 0.82 | 0.46 | 0.12 | 0.07 |
|  |  |  |  |  |  |  |  | Total | 46.3 |  |  |  |  |  |  |  |  |  |  |  |
| SPAD index |  |  |  |  |  |  |  |  |  |  |  |  |  |  |  |  |  |  |  |  |
|  |  | DJ |  | Pv04/32.8 |  | 5.1 |  |  | 13.1 |  | G/A |  | 3.38 |  | 0.90 | 0.89 | 0.68 | 0.88 | 0.93 | 0.93 |
|  |  |  |  | Pv11/32.2 |  | 6.0 |  |  | 16.9 |  | C/T |  | 5.54 |  | 0.97 | 1.00 | 0.77 | 0.96 | 0.95 | 0.98 |
|  |  |  |  |  |  |  |  | Total | 26.9 |  |  |  |  |  |  |  |  |  |  |  |
|  |  | MA |  | Pv10/15.2 |  | 4.5 |  |  | 19.7 |  | T/C |  | 3.38 |  | 0.62 | 0.75 | 0.73 | 0.69 | 0.76 | 0.57 |
| Adventitious root |  |  |  |  |  |  |  |  |  |  |  |  |  |  |  |  |  |  |  |  |
|  |  | DJ |  | Pv04/43.9 |  | 3.7 |  |  | 12.1 |  | A/C |  | 0.27 |  | 0.79 | 0.43 | 0.32 | 0.50 | 0.07 | 0.26 |
|  |  |  |  | Pv06/3.1 |  | 4.2 |  |  | 11.1 |  | G/A |  | 0.32 |  | 0.90 | 0.71 | 0.86 | 0.88 | 0.62 | 0.41 |
|  |  |  |  |  |  |  |  | Total | 22.7 |  |  |  |  |  |  |  |  |  |  |  |
|  |  | MA |  | Pv05/39.8 |  | 4.0 |  |  | 16.9 |  | A/T |  | 0.28 |  | 0.93 | 0.93 | 0.77 | 0.50 | 0.21 | 0.22 |
|  |  |  |  | Pv09/13.6 |  | 3.5 |  |  | 14.3 |  | T/C |  |  |  | 0.66 | 0.57 | 0.68 | 0.46 | 0.60 | 0.41 |
|  |  |  |  |  |  |  |  | Total | 28.5 |  |  |  |  |  |  |  |  |  |  |  |
| † The nucleotide with the positive effect (favorable allele) is represented first. | | | | | | | | | | | | | | | | | | | | |

| Table S4. List of potential candidate genes, associated with major significant loci, detected in flooded stress | | | | | | | | |
| --- | --- | --- | --- | --- | --- | --- | --- | --- |
| Trait† | Locus | Distance | *Phaseolus* gene | Best *Arabidopsis* hit | Pfam | Panther | Function | Promoter |
| GR | Pv01/42.4 | -34,410 | Phvul.001G163100 | AT3G07880.1 | RHO protein GDP dissociation inhibitor | RHO GDP-DISSOCIATION INHIBITOR | SCN1 | AP2/MYB |
|  | Pv02/36.9 | -37,379 | Phvul.002G209200 | AT5G64210.1 | Alternative oxidase | - | alternative oxidase 2 | MYB |
|  |  | 93,925 | Phvul.002G210200 | AT5G64260.1 | Phosphate-induced protein 1 conserved region | - | EXORDIUM like 2 | MYB/NAC/ WRKY |
|  | Pv02/41.0 | -19,788 | Phvul.002G243600 | AT1G78580.1 | Trehalose-phosphatase,  Glycosyltransferase family 20 | TREHALOSE-6-PHOSPHATE SYNTHASE | trehalose-6-phosphate synthase | MYB/NAC/ WRKY |
|  | Pv03/0.1 | -453 | Phvul.003G001700 | AT2G38770.1 | - | INTRON-BINDING PROTEIN AQUARIUS  DNA2/NAM7 HELICASE FAMILY | nucleoside triphosphate hydrolases superfamily protein.  EMBRYO DEFECTIVE 2765 | MYB/NAC/ WRKY |
| TW | Pv01/1.1 | 1,648 | Phvul.001G013100 | AT1G19600.1 | pfkB family carbohydrate kinase | SUGAR KINASE | pfkB-like carbohydrate kinase family protein | MYB/NAC/ WRKY |
|  |  | 36,537 | Phvul.001G013600 | AT1G75260.1 | - | - | oxidoreductases, acting on NADH or NADPH | MYB |
|  | Pv08/1.6 | 52,947 | Phvul.008G019600 | AT5G13330.1 | AP2 domain | - | Rap2.6L | MYB/NAC/WRKY |
|  | Pv10/2.8 | 34,252 | Phvul.010G018700 | AT2G44730.1 | - | - | Alcohol dehydrogenase transcription factor Myb/SANT-like family protein | AP2/B3/MYB/NAC/WRKY |
|  | Pv10/33.9 | 56,572 | Phvul.010G092300 | AT5G19790.1 | AP2 domain | - | RAP2.11 | MYB/NAC/ WRKY |
| SW | Pv01/1.2 | -1,693 | Phvul.001G014900 | AT4G34480.1 | Glycosyl hydrolases family 17,  X8 domain | - | carbohydrate metabolic process | AP2/MYB/NAC/WRKY |
|  | Pv07/7.7 | -14,968 | Phvul.007G080800 | AT1G43620.1 | Glycosyltransferase family 28 N-terminal domain,  UDP-glucoronosyl and UDP-glucosyl transferase | GLUCOSYL/GLUCURONOSYL TRANSFERASES | Glycosyltransferase superfamily protein | MYB |
|  | Pv08/55.5 | -1,277 | Phvul.008G241300 | AT1G73370.1 | Glycosyl transferases group 1,  Sucrose synthase | GLYCOSYLTRANSFERASE,  SUCROSE SYNTHASE 2-RELATED | carbohydrate metabolic process | MYB |
|  | Pv11/1.2 | -18,027 | Phvul.011G016300 | AT3G47420.1 | Major Facilitator Superfamily | SODIUM-DEPENDENT PHOSPHATE TRANSPORTERS | phosphate starvation-induced gene 3 | MYB/NAC/ WRKY |
|  |  | 110,788 | Phvul.011G017700 | AT3G13960.1 | WRC, QLQ | - | growth-regulating factor 5 | MYB |
|  | Pv11/4.3 | 12,840 | Phvul.011G050500 | AT5G57720.1 | B3 DNA binding domain | - | AP2/B3-like transcriptional factor family protein | NAC/WRKY |
| RW | Pv08/1.6 | 48,667 | Phvul.008G019600 | AT5G13330.1 | AP2 domain | - | Rap2.6L | MYB/NAC/WRKY |
| HL | Pv04/3.1 | 112,231 | Phvul.004G101900 | AT5G09280.1 | Pectate lyase | - | Pectin lyase-like superfamily protein | MYB |
|  | Pv04/42.2 | 49,465 | Phvul.004G143500 | AT1G73360.1 | Homeobox domain | - | homeodomain GLABROUS 11 | MYB |
|  | Pv04/24.9 | -96,255 | Phvul.004G092700 | AT1G29930.1 | Chlorophyll A-B binding protein | CHLOROPHYLL A/B BINDING PROTEIN | chlorophyll A/B binding protein 1 | MYB/NAC/WRKY |
|  |  | 43,372 | Phvul.004G092900 | AT2G34430.1 | Chlorophyll A-B binding protein | CHLOROPHYLL A/B BINDING PROTEIN | light-harvesting chlorophyll-protein complex II subunit B1 | - |
| SI | Pv04/41.2 | -37,434 | Phvul.004G134400 | AT4G09670.1 | Oxidoreductase family, Oxidoreductase family, NAD-binding Rossmann fold | OXIDOREDUCTASES | Oxidoreductase family protein | MYB/NAC/WRKY |
|  |  | -170,997 | Phvul.004G133400 | AT5G49890.1 | Voltage gated chloride channel, CBS domain | CHLORIDE CHANNEL | chloride channel C | MYB/NAC/WRKY |
|  | Pv08/23.6 | -795 | Phvul.008G140700 | AT4G01070.1 | UDP-glucoronosyl and UDP-glucosyl transferase | GLUCOSYL/GLUCURONOSYL TRANSFERASES | UDP-Glycosyltransferase superfamily protein | MYB/NAC/WRKY |
|  |  | -195,856 | Phvul.008G140100 | AT3G18990.1 | B3 DNA binding domain | - | AP2/B3-like transcriptional factor family protein | AP2/MYB/NAC/WRKY |
|  | Pv10/30.2 | 87,037 | Phvul.010G082000 | AT4G29890.1 | - | IRON-SULFUR DOMAIN CONTAINING PROTEIN, CHOLINE MONOOXYGENASE, CHLOROPLASTIC | choline monooxygenase, putative (CMO-like) | MYB/NAC/WRKY |
|  | Pv10/36.7 | 21,305 | Phvul.010G107400 | AT2G46240.1 | IQ calmodulin-binding motif,  BAG domain | - | BCL-2-associated athanogene 6 | MYB |
|  |  | 138,429 | Phvul.010G108000 | AT1G17420.1 | Lipoxygenase | - | lipoxygenase 3 | MYB/NAC/WRKY |
|  | Pv11/32.2 | -185,619 | Phvul.011G137200 | AT5G54250.1 | Cyclic nucleotide-binding domain | VOLTAGE AND LIGAND GATED POTASSIUM CHANNEL | cyclic nucleotide-gated cation channel 4 | MYB |
| AR | Pv04/43.9 | -1,648 | Phvul.004G157100 | AT3G02550.1 | - | - | LOB domain-containing protein 41 (LBD41) | NAC/WRKY |
|  | Pv05/38.5 | 2,893 | Phvul.005G159600 | AT1G72960.1 | Root hair defective 3 GTP-binding protein (RHD3) | GUANYLATE-BINDING PROTEIN | Root hair defective 3 GTP-binding protein (RHD3) | MYB |
|  | Pv07/45.1 | 3,133 | Phvul.007G212900 | AT2G47260.1 | WRKY DNA -binding domain | - | WRKY DNA-binding protein 23 | - |
|  | Pv09/35.3 | 9,453 | Phvul.009G240400 | AT3G54070.1 | Ankyrin repeat | Ankyrin repeat-containing protein | Ankyrin repeat family protein | MYB/WRKY |
|  | Pv10/0.8 | 26,504 | Phvul.010G005100 | AT5G01220.1 | Glycosyl transferases group 1 | GLYCOSYLTRANSFERASE | sulfoquinovosyldiacylglycerol 2 (SQD2) | MYB/NAC/WRKY |
|  | Pv11/0.7 | -1,510 | Phvul.011G010200 | AT3G52080.1 | Sodium/hydrogen exchanger family | - | cation/hydrogen exchanger 28 | AP2/B3/MYB/NAC |
| † Abbreviate name of the trait. TW: total weight, SW: shoot weight, RW: root weight, HL: hypochotyl length, SI: SPAD index, AR: adventitious rate | | | | | | | | |
